# Association Between Internet Use, Cognitive Function, and Globus Pallidus Volumes: A Study Among the Elderly in Chinese Communities

**DOI:** 10.3389/fpubh.2022.886974

**Published:** 2022-05-11

**Authors:** Wei Li, Ling Yue, Shifu Xiao

**Affiliations:** ^1^Department of Geriatric Psychiatry, Shanghai Mental Health Center, Shanghai Jiao Tong University School of Medicine, Shanghai, China; ^2^Department of Geriatric Psychiatry, Alzheimer's Disease and Related Disorders Center, Shanghai Jiao Tong University, Shanghai, China

**Keywords:** internet use, cognition, elderly, MRI, globus pallidus

## Abstract

**Background:**

Previous studies have linked internet use with several beneficial outcomes for brain health, but there is little data on this among older Chinese.

**Objective:**

The goal of this study was to explore the association between internet use and cognitive impairment and to explore the possible mechanisms by which internet use prevents cognitive decline.

**Methods:**

The current study consisted of two cohorts: one from the China Longitudinal Aging Study (CLAS), which included 610 older adults with mild cognitive impairment (MCI), 192 with dementia, and 2,218 healthy older adults; the second cohort included 39 healthy adults from the Shanghai brain health foundation (SHBHF2016001), who underwent T1 cranial magnetic resonance imaging at baseline, from which their volumes of the hippocampus, amygdala, and globus pallidus were calculated. Moreover, they were also followed up for 1 year. Through standardized questionnaires, detailed general demographic information and internet use information was obtained. The cognitive diagnosis of each participant was made by attending psychiatrists at baseline, and their overall cognitive function was assessed by the mini-mental state examination (MMSE) and Montreal Cognitive Assessment (MoCA).

**Results:**

In cohort 1, after controlling age, gender, smoker, tea drinker, take exercise, diabetes, and hypertension, we found that internet use was associated with MCI (*P* = 0.015, OR = 0.478, 95% CI: 0.264~0.867), but not for dementia (*P* = 0.205, OR = 0.393, 95% CI: 0.093~1.665). In cohort 2, we found that the globus pallidus volume of internet users was significantly larger than that of non-users, and the MMSE change score was lower (*P* < 0.05). The results of partial correlation analysis (controlled for sex, age and education) showed that the change of MMSE value was positively correlated with the volume of left globus pallidus (*r* = 0.547, *P* = 0.004).

**Conclusions:**

Internet use might be a protective factor for mild cognitive impairment among the Chinese elderly, and it may prevent a decline in cognitive decline by affecting the volume of the globus pallidus.

## Introduction

Dementia, the most serious form of cognitive impairment, is the leading cause of disability among the elderly and currently affects nearly 50 million people worldwide. If the prevalence of dementia remains unchanged, that number is expected to increase to more than 130 million by 2050 (1). Caring for people with dementia is a heavy financial and emotional burden on both families and society, and the yearly per-person cost attributable to dementia ranged from $41,689 to $56,290 (2). Since there are no commercially available disease-modifying therapies, there is a growing emphasis on strategies to prevent dementia (3). Overall, there is growing evidence supporting 12 potentially modifiable risk factors for dementia: smoking, obesity, depression, diabetes, hypertension, air pollution, less education, physical inactivity, hearing impairment, low social contact, traumatic brain injury, and excessive alcohol consumption. Together, these 12 modifiable risk factors account for about 40 percent of dementia worldwide, so dementia could theoretically be delayed or prevented (4).

Internet use, including web browsing, daily office, and exchanging e-mails, is thought to be crucial for dealing with various everyday tasks nowadays. In the past few years, there has been a marked increase in the use of computers and the internet by the elderly; for example, a whopping 56 percent of seniors aged 65–74 had access to the internet in 2011, and that figure rose to 71 percent in 2015 (5). Accumulating research suggests that puzzle games can help prevent cognitive decline (6–9), but only a few studies have explored the link between internet use and cognitive impairment. For example, d' Orsi et al. (5) followed 8,238 dementia-free participants for 4 years (aged 50 years or older), and found that those baseline internet users were associated with a 40% reduction in dementia risk (HR = 0.60; CI: 0.42–0.85; *P* < 0.05); Benjamin David Williams et al. followed 3,937 older British people over the age of 50 for 7 years, and also found that internet/email use was associated with reduced risk of cognitive impairment (10). However, they were unable to explain the potential causal mechanisms underlying this association.

So far, there is no longitudinal study on the relationship between internet use and cognitive function in China. In order to fill this gap, and to reveal the possible mechanism of internet use in preventing cognitive decline, we included a national cross-sectional study and a 1-year longitudinal follow-up study. Our hypothesis was that internet use can help prevent cognitive decline, and the mechanism might be related to its influence on cognition-related brain regions.

## Methods

### Data Sources

The current cross-section study was derived from the China Longitudinal Aging Study (CLAS) (11) and Shanghai brain health foundation (SHBHF2016001) (12), which have both been described in detail in our previous study (12–14). The first cohort included a total of 3,020 community participants aged 60 years or older, including 610 mild cognitive impairment (MCI), 192 dementia, and 2,218 normal controls. All participants underwent a screening process that included physical and neurological examinations, medical history, and cognitive assessments. All participants met the following requirements: (1) aged 60 or more; (2) without severe medical conditions, such as cancer, infections; (3) without serious mental illness, such as schizophrenia and severe depression; (4) be willing to cooperate. Their diagnosis was carried out by experienced psychiatrists, and the diagnosis of MCI was based on Petersen's criteria (15), while the diagnosis of dementia was based on DSM IV.

A total of 49 people over 60 years of age (mean age: 64.78 ± 4.597; average years of education: 11.43 ± 2.708; male: *n* = 40, 81.6%) were included in cohort 2. They were divided into internet users (*n* = 19) and non-internet users (*n* = 30) based on whether they were internet users or not, and followed for 1 year (from 2013 to 2014). All the subjects had normal cognitive function at baseline and underwent a T1 magnetic resonance scan. The inclusion criteria were as follows: (1) Han Chinese; (2) ≥60 years old; (3) absence of mild cognitive impairment (MCI) or dementia; (4) the Mini-Mental State Examination (MMSE) score was ≥27; without serious physical or mental illness; (5) able to complete the study (14). Exclusion criteria were (1) <60 years old; (2) suffered from severe visual or hearing impairment; (3) refusal of the participants or their guardians to participate in the study.

The project was approved by the Ethics Committee of the Shanghai Mental Health Center and all the subjects had signed an informed consent before the study was initiated. The whole study was conducted in accordance with the principles of Declaration of Helsinki.

### Internet Use Measurement

Internet use was defined as whether respondents used the internet and/or email (16). The question “Whether you use the internet or email, and how often do you use the internet?” was used in the interview at baseline. People were classified as not using the internet reported that they used it less than once every 3 months or never. On top of that, we also collected how long they had been using the internet (5).

### Cognitive Assessment

Mini-Mental State Examination was used to measure the subjects' overall cognitive function (17). This screening test consists of 30 items that measure multiple cognitive domains including naming, attention, calculation, abstract, orientation, memory, visual space, as well as language function, and it is one of the most common cognitive assessment tools. In addition to MMSE, the Montreal Cognitive Assessment (MoCA) was also used in the current study. Compared with MMSE, MOCA is more sensitive and specific for screening for MCI and is more likely to capture changes in cognitive function. Therefore, in the current study, we used both MMSE and MoCA to assess cognitive changes at baseline and follow-up (cohort 2).

### MR Image Acquisition and Processing

All the subjects had completed T1 structural magnetic resonance scans at baseline. The images were acquired by using a Siemens Magnetom Verio 3.0T scanner (Siemens, Munich, Germany), and the parameters were as follows: TE = 2.98 ms, TR = 2,300 ms, slice thickness, 1.2 mm, flip angle, 9°, matrix size, 240 × 256, field of view (FOV), 240 × 256 mm, and the number of slices, 176 (14). All the sMRI data were processed using Clinica in FreeSurfer v6.0, including spatial registration, cortical thickness estimation, extraction of cortical surface segmentation of the subcortical structures, and parcellation into 46 global structures (12). Based on previous studies, we used the hippocampus, amygdala, and globus pallidus as our core research brain area (18).

### Covariates

The following data, such as age, gender, education, daily living information (hobby, physical activities, sleeping patterns, dietary preferences, smoking history, and consumption of tea and alcohol) as well as disease related information (such as hypertension, and diabetes) were also collected by standardized questionnaire.

### Data Analysis

Continuous variables were expressed as mean ± SD, while categorical variables were expressed as frequencies (%). A single factor ANOVA and Chi-square tests were used to compare continuous and categorical variables in the MCI group, dementia group, and normal control group, respectively. Then a multivariate logistic regression model was used to investigate the association between internet use and cognitive impairment, controlling for variables that differed between the three groups, such as age, gender, smoker, tea drinker, take exercise, diabetes, and hypertension (Cohort 1). In Cohort 2, a single sample Kolmogorov–Smirnov test was used to test whether data conforms to normal distribution. Then independent sample *t*-test and Kruskal–wallis *H* were used to compare the normal data and non-normal data between the internet user group and the non-internet user group, respectively. Finally, partial correlation analysis (controlled for age, sex, and education) was used to investigate the association between MMSE score changes and cognition-related brain regions. Two-tailed tests were used at a significance level of *P* < 0.05 for all analyses. The data was analyzed using SPSS 22.0 (IBM Corporation, Armonk, NY, USA).

## Results

### The General Demographic Information of the Study Population (Cohort 1)

Table 1 shows the differences in general demographic information among the three groups (MCI, Dementia, and Normal). The proportion of internet users was significantly higher in the normal elderly (15.1%) than in the MCI (5.6%) and dementia (2.3%) groups. In addition, there were also statistical differences in age, gender, smoker, tea drinker, take exercise, diabetes, and hypertension among the three groups, with *P* values of <0.001, <0.001, 0.009, <0.001, <0.001, =0.001, and =0.006, respectively. Moreover, MMSE and MoCA scores were also statistically different among the three groups (*P* < 0.001).

**Table 1:** Comparison of baseline general demographic and cognitive characteristics among different cognitive populations.

**Variables**	**MCI (*n* = 610)**	**Dementia (*n* = 192)**	**Normal (*n* = 2,218)**	***F*** **or *X*^2^**	***P*** **-Value**
Age, years	73.85 ± 8.238	78.83 ± 7.538	70.10 ± 7.531	145.759	<0.001^*^
Education, years	6.68 ± 3.225	6.47 ± 3.008	6.83 ± 3.505	1.314	0.269
Male, *n* (%)	233 (38.2)	71 (37.0)	1,074 (48.4)	26.347	<0.001^*^
Smoker, *n* (%)	147 (24.1)	43 (22.4)	650 (29.3)	9.460	0.009^*^
Drinker, *n* (%)	119 (19.5)	31 (16.1)	475 (21.4)	3.646	0.162
Tea drinker, *n* (%)	220 (36.1)	57 (29.7)	1,116 (50.3)	61.390	<0.001^*^
Take exercise, *n* (%)	389 (63.8)	92 (47.9)	1,689 (76.1)	94.345	<0.001^*^
Diabetes, *n* (%)	108 (17.7)	49 (25.5)	346 (15.6)	13.136	0.001^*^
Hypertension, *n* (%)	294 (48.2)	112 (58.3)	1,029 (46.4)	10.244	0.006^*^
Internet use	17 (5.6)	2 (2.3)	218 (15.1)	29.750	<0.001^*^
MMSE	22.38 ± 5.729	13.97 ± 7.412	26.80 ± 3.508	877.044	<0.001^*^
MoCA	16.72 ± 6.150	9.10 ± 6.250	22.79 ± 5.179	724.147	<0.001^*^

*MMSE, Mini-Mental State Examination; MoCA, Montreal Cognitive Assessment*.

* *P < 0.05*.

### Possible Factors Associated With MCI and Dementia by Multivariate Logistic Regression Models (Cohort 1)

Based on previous statistical conclusions and clinical experience, internet users, age, gender, smokers, tea drinkers, take exercise, diabetes, and hypertension were included in the multivariate logistic regression models. We found that internet user was associated with MCI (*P* = 0.015, OR = 0.478, 95% CI: 0.264~0.867), but not for dementia (*P* = 0.205, OR = 0.393, 95% CI: 0.093~1.665). Table 2 presents the results.

**Table 2:** Association of internet use with cognitive change (MCI or dementia).

**Variables**	***B***	**SE**	**Wald**	**df**	***P*** **-Value**	**OR**	**95% confidence interval**
**MCI**							
Age	0.079	0.009	79.167	1	<0.001^*^	1.082	1.063–1.101
Male	−0.335	0.166	4.048	1	0.044^*^	0.716	0.516–0.991
Smoker	0.159	0.185	0.733	1	0.392	1.172	0.815–1.686
Tea drinker	−0.267	0.147	3.303	1	0.069	0.766	0.574–1.021
Take exercise	−0.131	0.152	0.743	1	0.389	0.877	0.651–1.182
Diabetes	0.184	0.182	1.021	1	0.312	1.202	0.842–1.716
Hypertension	−0.100	0.139	0.513	1	0.474	0.905	0.689–1.189
Internet use	−0.738	0.304	5.897	1	0.015^*^	0.478	0.264–0.867
**Dementia**							
Age	0.134	0.016	71.746	1	<0.001^*^	1.143	1.108–1.179
Male	−0.258	0.285	0.819	1	0.365	0.773	0.442–1.351
Smoker	0.178	0.326	0.297	1	0.586	1.195	0.630–2.265
Tea drinker	−0.431	0.263	2.679	1	0.102	0.650	0.388–1.089
Take exercise	−0.907	0.243	13.917	1	<0.001^*^	0.404	0.251–0.650
Diabetes	0.633	0.284	4.960	1	0.026^*^	1.883	1.079–3.287
Hypertension	0.414	0.252	2.705	1	0.100	1.513	0.924–2.478
Internet use	−0.935	0.737	1.609	1	0.205	0.393	0.093–1.665

*MCI, mild cognitive impairment*.

* *P < 0.05*.

### The Connection Between Internet Users and Brain Structure (Cohort 2)

**Figure 1:**
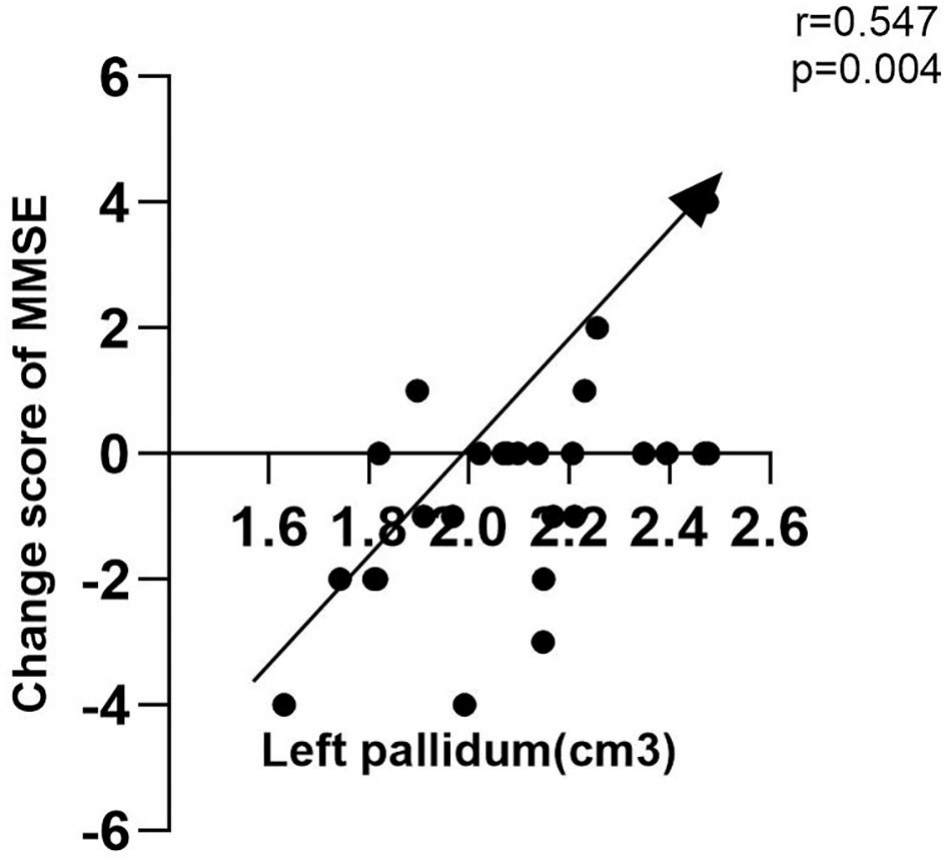
Association between MMSE change score and globus pallidus volumes.

To explain the possible mechanisms by which internet users affect cognition, we added structural magnetic resonance data. Based on whether they had a habit of using the internet, these 49 people were divided into an internet user group (*n* = 19) and non-internet users (*n* = 30) group and followed for 1 year. There was no statistical difference (*P* > 0.05) in age, education, gender, smoker, drinker, tea drinker, take exercise, diabetes, hypertension, whole brain volume, right amygdala, baseline MMSE, follow up of MMSE, baseline MoCA, follow up of MoCA, and MoCA change score between the two groups. However, the volume of left hippocampus, right hippocampus, left amygdala, left globus pallidum, and right globus pallidum in the internet users group were all larger than those in the non-internet users group, while the MMSE change score in the internet users group was significantly smaller than that in the non-internet users group (*P* < 0.05). Table 3 presents the results. Then by using partial correlation analysis (controlled age, gender, and education), we found that the volume of left globus pallidus was positively correlated with the change value of MMSE (*r* = 0.547, *P* = 0.004). Figure 1 presents the results.

**Table 3:** Comparison of baseline brain structure and cognitive function between internet users and non-internet users.

**Variables**	**Internet users (*n* = 19)**	**Non-internet users (*n* = 30)**	***X***^2^ **or *t* or *Z***	***P*** **-Value**
Age, years	64.58 ± 4.948	64.90 ± 4.444	−0.236	0.815
Education, years	11.53 ± 2.695	11.37 ± 2.760	0.199	0.843
Male, *n* (%)	15 (78.9)	25 (83.3)	0.149	0.720
Smoker, *n* (%)	8 (42.1)	14 (46.7)	0.098	0.777
Drinker, *n* (%)	6 (31.6)	8 (26.7)	0.138	0.754
Tea drinker, *n* (%)	13 (68.4)	14 (46.7)	2.225	0.155
Take exercise, *n* (%)	10 (52.6)	21 (70.0)	1.510	0.242
Diabetes, *n* (%)	4 (21.1)	6 (20.0)	0.008	1.000
Hypertension, *n* (%)	8 (42.1)	13 (43.3)	0.007	1.000
Whole brain volume, cm^3^	1,573.63 ± 117.264	1,524.69 ± 92.251	1.627	0.110
Left hippocampus, cm^3^	4.034 ± 0.410	3.747 ± 0.342	2.648	0.011^*^
Right hippocampus, cm^3^	4.251 ± 0.377	3.971 ± 0.361	2.602	0.012^*^
Left amygdala, cm^3^	1.718 ± 0.242	1.579 ± 0.185	2.260	0.028^*^
Right amygdala, cm^3^	1.877 ± 0.288	1.735 ± 0.234	1.889	0.065
Left globus pallidus, cm^3^	2.167 ± 0.256	2.034 ± 0.167	2.210	0.032^*^
Right globus pallidus, cm^3^	2.123 ± 0.206	1.938 ± 0.173	3.374	0.001^*^
Baseline MMSE	28.53 ± 1.712	28.77 ± 1.305	−0.556	0.581
Follow up of MMSE	28.25 ± 1.658	27.82 ± 1.879	0.631	0.533
MMSE change score	0.000 ± 2.044	−1.177 ± 1.237	−2.235	0.033^*^
Baseline MoCA	26.16 ± 2.630	25.87 ± 2.623	0.378	0.707
Follow up of MoCA	25.42 ± 3.088	25.82 ± 3.187	−0.343	0.734
MoCA change score	0.000 ± 3.104	0.824 ± 3.067	−0.709	0.485

*MMSE, Mini-Mental State Examination; MoCA, Montreal Cognitive Assessment*.

* *P < 0.05*.

## Discussion

Older adults who bridge the digital divide have been proved to experience a series of positive outcomes compared to those who are not online, including higher feelings of empowerment, wellbeing and self-efficacy (19). Despite research on the benefits of internet use among older adults is encouraging, there is little data directly linking internet use to the risk of future cognitive impairment among older adults. To solve the above problems, we adopted two large cohort studies to explore the relationship between internet use and cognitive impairment, and we found that (1) internet use was associated with MCI, but had nothing to do with dementia, (2) the old man often use the internet had a greater volume of globus pallidus, while smaller changes in MMSE score; (3) the volume of globus pallidus on the left was positively correlated with MMSE change value. These results suggested that internet use might prevent cognitive decline by affecting the volume of the globus pallidus.

In our first large cross-sectional cohort study, we examined the association between internet use and cognitive functioning among older adults in the community (including 610 older adults with MCI, 192 with dementia and 2,218 normal elderly adults). We found that the percentage of people with dementia and MCI using the internet was significantly lower than the normal elderly. After controlling for gender, age, education, and disease, we found that internet use was still associated with MCI, but not dementia. In Su et al. (20) study, they found that hobbies, such as reading, surfing the internet, were protective factors for MCI. In Wang et al.'s (21) study, they found that hobbies could help prevent MCI. In Shimada et al.'s (22) study, they found that participating in hobbies or sports activities would help reverse MCI. Since these studies did not specifically examine the link between internet use and MCI, we could say that we were the first to find that internet use might help reduce the risk of MCI. But we have to point out, studies have shown that heavy use of electronic devices (i.e., more than 2–3 h/day exposure to electronic media including computers, television, and mobile devices such as tablets, smartphones, and laptops) cloud also increase the risk of cognitive impairment (23). Therefore, we recommend that the elderly should not spend more than 2 h on the computer on a daily basis.

To further explore the possible mechanism by which the internet prevents cognitive decline, we added t1-phase cranial magnetic resonance (including volume of hippocampus, amygdala, and globus pallidus) in the second clinical cohort, and it included 49 healthy elderly people who were followed for 1 year. We finally found that the internet users had larger globus pallidus volume and that the volume on the left side was positively correlated with changes in MMSE scores, suggesting that the globus pallidus volume might be a protective factor for cognitive decline. Since this was the first study to explore the relationship between internet use and cognition-related brain regions, we could not tell if our findings were consistent with those of others.

The globus pallidus (GP) is a major component of the basal ganglia (BG) and can be divided into the internal segment (GPi, output nucleus) and the external segment (GPe, intermediate nucleus). It communicates with widespread cortical areas that support various functions, including cognition, motivation, and action (24). Previous studies suggest that deep brain stimulation (DBS) of the globus pallidus interna (GPi) can reduce motor symptoms in patients with Parkinson's disease (PD) and improve their cognitive function and quality of life (25–27). It is also considered as a treatment option for Huntington's disease (HD) patients with severe pharmacologically refractory chorea (28). Moreover, changes in mitochondrial morphology in the globus pallidus can also be observed in Alzheimer's disease (29). Therefore, combined with our current findings and the basis of previous studies, we speculated that deep stimulation of the globus pallidus may also improve the cognitive symptoms of dementia patients, but the above hypothesis needs to be further verified by larger studies.

We must admit that there are two limitations to our study. First, information on internet use is obtained through self-report rather than objective assessment, so there is the possibility of recall bias. Secondly, since this first cohort was only a cross-sectional study, it couldn't indicate the causal effect between internet use and MCI. Thirdly, the short follow-up time was also the main limitation of this study.

## Conclusions

In conclusion, internet usage among Chinese elderly is associated with a lower incidence of mild cognitive impairment, and internet use may prevent cognitive decline by affecting the globus pallidus volume.

## Data Availability Statement

The original contributions presented in the study are included in the article/supplementary material, further inquiries can be directed to the corresponding authors.

## Ethics Statement

The studies involving human participants were reviewed and approved by the Ethics Committee of the Shanghai Mental Health Center. The patients/participants provided their written informed consent to participate in this study.

## Author Contributions

WL and LY contributed to the study concept and design. SX analyzed the data and drafted the manuscript. All authors read and approve the final manuscript.

## Funding

This study was supported by grants from the clinical research center project of Shanghai Mental Health Center (CRC2017ZD02), Clinical Research plan of SHDC (SHDC2020CR1038B), the Cultivation of Multidisciplinary Interdisciplinary Project in Shanghai Jiaotong University (YG2019QNA10), curriculum reform of Medical College of Shanghai Jiaotong University, and the Feixiang Program of Shanghai Mental Health Center (2020-FX-03 and 2018-FX-05), the National Natural Science Foundation of China (82101564 and 82001123), Chinese Academy of Sciences (XDA12040101), Shanghai Clinical Research Center for Mental Health (SCRC-MH and 19MC1911100), the Shanghai Science and Technology Committee (20Y11906800), and Shanghai Brain Health Foundation (SHBHF2016001).

## Conflict of Interest

The authors declare that the research was conducted in the absence of any commercial or financial relationships that could be construed as a potential conflict of interest.

## Publisher's Note

All claims expressed in this article are solely those of the authors and do not necessarily represent those of their affiliated organizations, or those of the publisher, the editors and the reviewers. Any product that may be evaluated in this article, or claim that may be made by its manufacturer, is not guaranteed or endorsed by the publisher.
